# Students’ perceptions on race in medical education and healthcare

**DOI:** 10.1007/s40037-020-00645-6

**Published:** 2021-01-07

**Authors:** Guan Hui Tricia Lim, Zeluleko Sibanda, Joshua Erhabor, Soham Bandyopadhyay, The Neurology and Neurosurgery Interest Group

**Affiliations:** 1 University College London Medical School, University College London, London, UK; 2 Keele University, School of Medicine, Keele, UK; 3 University of Exeter, School of Medicine, Exeter, UK; 4 Oxford University Global Surgery Group, Nuffield Department of Surgical Sciences, University of Oxford, Oxford, UK

**Keywords:** Medical education, Race, Medical students, Institutional racism

## Abstract

Major racial disparities continue to exist in our healthcare education, from the underrepresentation of ethnic minorities when teaching about clinical signs to health management in primary and secondary care. A multi-centre group of students discuss what needs to change in medical education to cultivate physicians who are better prepared to care for patients of all backgrounds. We argue that the accurate portrayal of race in medical education is a vital step towards educating medical students to consider alternative explanations to biology when considering health inequities.

## Background

Two events have so far defined 2020: the COVID-19 (Coronavirus disease 2019) pandemic and the death of George Floyd. COVID-19 refers to an ongoing global pandemic caused by a novel coronavirus (SARS-CoV-2) [1, 2]. A key aspect of this pandemic has been the disproportionately high COVID-19 mortality rate in individuals from Black, Asian and Minority Ethnic (BAME) communities [3, 4]. George Floyd was an African-American Black man killed by the police during an arrest [5]. His death was partly contributed to by a delayed medical response [6]. Following Mr. Floyd’s murder, the initial autopsy conducted by a government agency stated that Mr. Floyd was “high on fentanyl and had recently used methamphetamine at the time of his death”, and claimed there were “no physical findings that support a diagnosis of traumatic asphyxia or strangulation” [7]. This finding was successfully challenged, and it was found that Mr. Floyd had died due to asphyxiation from sustained pressure exerted by a police officer on scene [8]. BAME individuals being disproportionately affected by COVID-19 [3, 4], the use of excessive force by police officers against Black suspects [9], and the conflicting autopsy result can be attributed to a type of institutionalised discrimination known as institutional racism [10, 11].

Institutionalised discrimination is discrimination that is encoded into the operating procedures, policies, laws, and/or objectives of society and its institutions as a whole. These unjust biases embedded in normal practice lead to the mistreatment of an individual or group of individuals. Institutional racism as a type of institutionalised discrimination was first described in 1967 [10]. Since then, institutional racism has been formally defined as “the collective failure of an organization to provide an appropriate and professional service to people because of their colour, culture, or ethnic origin. It can be seen or detected in processes, attitudes, and behaviour that amount to discrimination through prejudice, ignorance, thoughtlessness, and racist stereotyping which disadvantage minority ethnic people” [11]. However, the concept of institutional racism is not appreciated or accepted by all. Following the publication of the higher COVID-19 mortality rate in BAME communities, a government official in the UK claimed that BAME individuals are “not taking the pandemic seriously” and suggested they are to blame for the spread of COVID-19 [12]. Not only does this perspective overlook the societal and structural factors—housing status, types of job, and modes of travel to name a few—that have predisposed BAME individuals to increased risk, but it is this type of thoughtlessness and racist stereotyping mentioned above that perpetuates institutional racism [13].

Institutional racism in healthcare is not a phenomenon unique to 2020. In 1992, Hutchinson described how public health initiatives aimed at black communities were slow to develop and insensitive to their needs [14], thereby making them more likely to fail. The *British Medical Journal* has also devoted an issue of their journal towards highlighting issues that affect doctors and patients from ethic minority backgrounds, and as such the need to tackle institutional racism in healthcare settings [15, 16]. Despite its importance and its demonstrable role in perpetrating health inequalities, there has been a noticeable lack of attention on if and how institutional racism could be perpetuated by medical schools. This is a broad topic that includes admission practices, classroom dynamics, diversity within faculty, content of taught material and the attitudes of teachers, to name but a few. To cover all of these topics sufficiently would be beyond the scope of this article. Instead, this article will aim to showcase how race is conceptualised, and how it is taught and examined during medical school.

## Is race a biological concept?

The use of genetic language when discussing race in the context of public health is thought to have directly contributed to the historical exploitation of Black lives for major medical discoveries [17]. Although medicine has come a long way since then, race is still often operationalised as a biological concept in medical school teaching [18] and presented as an independent risk factor for various diseases. This attribution of outcomes to race can contribute to bias and unequal care. The imprecise use of race as a proxy for pathology is an aspect of institutional racism, as it perpetuates the misunderstanding of race as solely a biological characteristic rather than a social construct [19–21]. Racial groups are not distinct homogenous genetic blocks. In fact, the genetic differences within each race far exceeds those between races [22].

An argument against the above is the high incidence of sickle cell disease (SCD)—a hereditary disease—in Black individuals [23]. SCD is frequently described as “an autosomal recessive disease that primarily affects persons of African ancestry” [24]. Within media and research, SCD has also commonly been portrayed as a “Black disease” [25]. Without context and deeper understanding about this condition, it is easy to put this down to coincidental genetic differences. However, this downplays the protective factor of the sickle haemoglobin (HbS) allele against malaria, therefore diminishing the evolutionary survival advantage SCD confers in malaria endemic regions. In other words, in malaria-infested areas, individuals with one HbS allele were less likely to die from malaria. This survival advantage increased the percentage of individuals in that area with the HbS allele. It so happens that malaria is endemic in West and Central Africa [26], regions in which the majority population is Black. Therefore, the gene is not associated with race, but rather with a biologic disease: malaria. The fact that the HbS allele is not solely found in Black populations [27] is evidence enough that genetic ancestry cannot be reduced into racial categories. It is essential that when topics like this are taught to medical students, social contextualisation—such as that provided above—occurs. Teaching without the contextualisation may leave students thinking that inherent differences exist between individuals of different races.

Currently, the inaccurate portrayal of race as a biologic datapoint has been legitimised to such an extent that normal physiological differences that are more prevalent in BAME individuals are now included in medical nomenclature, for example benign ethnic neutropenia [28, 29]. The risk of continuing to teach race as only a biological concept is that healthcare professionals may be being taught to use race in their clinical practice, which can lead to serious medical errors [30] for two reasons. The first is healthcare professionals are often unable to correctly establish an individual’s race independently [18, 31]. The second is patients may receive delayed or missed diagnoses if they present outside of simplified racial paradigms [32–35].

## Existing misconceptions—a legacy of the past

Our knowledge is built on our predecessors, and with their legacy we inherit both their wisdom and their errors. Several outmoded and unsubstantiated theories from the past have survived the tides of time and still haunt medical curriculums.

### Perception and assessment of pain in BAME individuals

Hailed as “the father of modern gynaecology”, Dr James Marion Sims conducted surgical experiments on enslaved Black women without anaesthesia, out of the false assumption that Black people did not feel as much pain. Unfortunately, this harmful stereotype still exists within medicine [17]. Over a century later in 2016, Hoffman showed that over half of their sample of American medical students and residents endorsed false viewpoints about biological differences between Black and white patients’ pain tolerance, deeming that Black patients may have a higher pain tolerance because “Black people’s skin is thicker than white people’s skin” [36]. This results in a flawed assessment of pain perception and treatment in Black patients. In 2009, Anderson found that Black patients are more likely to have their pain underestimated in comparison to white patients [37]. In fact, Black patients are less likely to be given analgesia or receive the same volume of analgesia when compared to white patients [37]. One reason why such bias remains prevalent amongst students and healthcare providers may be that existing educational materials continue to reinforce outmoded, harmful ways of thinking. A nursing textbook entitled “Nursing: A Concept-Based Approach to Learning” contained a section aimed at explaining cultural differences in response to pain. This section included statements such as “Jews may be vocal and demand assistance. They believe pain must be shared and validated by others” [38]. These assumptions rely on stereotyped opinions rather than evidence-based medicine; they not only encourage prejudiced pain assessment but also perpetuate racial and ethnic stereotypes. There is an urgent need to assess and update educational material like the above to ensure information is grounded in evidence-based medicine. This could be achieved via discussions with patients, researchers and healthcare professionals with interest in BAME issues.

### Racial “adjustment” in physiological measures

Medical students are taught that physiological measures such as spirometry values [39] and glomerular filtration rates (GFR) [40] should have a racial “adjustment”. This stemmed from the modification of diet in renal disease (MDRD) study that reported that Black people had a higher measured GFR for the same given creatinine level [40]. This association was explained by the assertion, without physiological evidence, that muscle mass varies by race [41]. This gained widespread acceptance due to the large number of individuals enrolled in the study and the fact that their GFR was directly measured. However, the study only involved people with chronic kidney disease (CKD) and therefore was not representative of the general population. As a consequence, a racial adjustment factor was introduced that increased the estimated GFR (eGFR) for a Black person for a given creatinine level (as Black people are assumed to have more muscle, and thus have higher creatinine levels). Compared to a white man of the same age, weight, and serum creatinine level, the eGFR of a Black man would be higher, suggesting that Black people have “better kidney function” than individuals of other races. As a result, Black patients must meet higher creatinine thresholds of kidney function in order to be diagnosed with kidney disease. Therefore, the higher incidence of end stage renal disease in Black patients may not be due to genetic causes, but because of the incorrect use of race as a proxy in GFR calculations, delaying diagnosis of CKD [42]. There is now increasing evidence that a racial adjustment factor does not always contribute to more accurate results on eGFR [43]. It is vital that medical students are made aware not just of the existence of these racial adjustment factors, but of the assumptions underlying the adjustments and the consequences if those assumptions are incorrect.

### The lack of race teaching

From an early stage in medical training, students are taught that diseases affect patients differently. However, there is a lack of emphasis placed on learning how to recognise different clinical presentations in patients from BAME backgrounds. Examples of this systemic lack of representation are seen in the teaching of dermatological signs or acute signs of cyanosis, where students are primarily shown how particular clinical signs present in white patients [44]. This lack of diversity in teaching is apparent in textbooks and in pre-clinical lecture presentations [45–47]. This is particularly stark in scoring systems such as APGAR (appearance, pulse, grimace, activity, respiration) where the category of appearance is scored by range in colour from pink to blue [48]. These dermatological differences are more visible in babies with white skin, with no official guide on how to recognise signs in BAME children with darker skin tones. Recognising this, innovative medical students such as Malone Mukwende have produced resources such as the “Mind the Gap” handbook which highlights clinical dermatological signs in BAME individuals [49]. Training modules and updated guides may be a practical improvement that could help reduce health inequalities [50]. To ensure our ability to eliminate these health inequalities, we need to address the issue as early as pre-clinical education with a clear focus on socio-economic aspects, improved representation, and systemic racism in healthcare.

### Race in exams

There needs to be a move away from using race in questions as a determining diagnostic hint for establishing the “most likely” diagnosis. Question banks that instil this form of pattern recognition through hundreds, even thousands, of practice questions encourage students to rely primarily on racial assumptions [51]. This risks oversimplifying race as purely a visual diagnostic concept and could lead to medical errors and delayed diagnosis [32–35]. In addition, the utilisation of racial categories as risk factors and pathological markers enforces the misunderstanding that race is solely a biological concept, which may fortify racial bias and stereotyping. Nonetheless, we appreciate that considering epidemiological prevalence is important in clinical practice, and hence we propose that an alternative approach is to address race in epidemiological questions, where it can be contextualised and the complex reality of inequality as it pertains to race explored.

## Conclusion

Race is a term often used in biomedical research, epidemiology, medical practice and education. However, the appropriate use of race in medicine remain elusive and controversial. The framing of race as a biological concept is problematic because race is not a biological category, and there are wider social and structural determinants of disease. The accurate portrayal of race in medical education is a vital step towards training medical students to consider alternative explanations to biology when considering health inequities. We hope the recommendations provided here (Box 1) will facilitate medical school faculty members and students to portray race in a way that cultivates physicians who are better prepared to care for patients of all backgrounds and tackles the perpetuation of institutional racism in healthcare.

### Box 1

**Issues**

1. Race is incorrectly operationalised as a biological concept, and attribution of outcomes to race can lead to medical errors.
2. Existing educational materials may reinforce institutional racism within medical education.
3. Using race in question banks encourages students to rely primarily on racial assumptions.

**Recommendations**

1. Provide social contextualisation when discussing incidence and prevalence of diseases among different races.
2. Assess and update educational material to remove stereotypes and ensure information is grounded in evidence-based medicine.
3. Educate medical students on the assumptions underlying racial adjustment factors.
4. Create training modules and guides on how clinical signs may manifest differently in individuals of different races.
5. Ensure that race is not used purely as a visual diagnostic concept in exam questions.
